# Novel design of cryptographic architecture of nanorouter using quantum-dot cellular automata nanotechnology

**DOI:** 10.1038/s41598-024-61260-7

**Published:** 2024-05-08

**Authors:** Sankit Kassa, Jadav Chandra Das, Vijay Lamba, Debashis De, Bikash Debnath, Saurav Mallik, Mohd Asif Shah

**Affiliations:** 1 Symbiosis Institute of Technology, Symbiosis International Deemed University, Pune, India; 2 Department of Information Technology, Maulana Abul Kalam Azad University of Technology, West Bengal, Haringhata, West Bengal 741249 India; 3 Department of Computer Science and Engineering, Maulana Abul Kalam Azad University of Technology, West Bengal, Haringhata, West Bengal 741249 India; 4 Department of Information Technology, Amity University, Kolkata, West Bengal India; 5 Department of Environmental Health, Harvard T H Chan School of Public Health, Boston, MA 02115 USA; 6 Department of Economics, Kebri Dehar University, Jigjiga, Somali 250, Ethiopia; 7 Division of Research and Development, Lovely Professional University, Phagwara, Punjab 144001, India

**Keywords:** Majority gate cryptography, Quantum-dot cellular automata (QCA), Encryption, Nanorouter, Decryption, Energy science and technology, Engineering, Materials science, Nanoscience and technology, Physics

## Abstract

The article introduces a revolutionary Nanorouter structure, which is a crucial component in the Nano communication regime. To complete the connection, many key properties of Nanorouters are investigated and merged. QCA circuits with better speed and reduced power dissipation aid in meeting internet standards. Cryptography based on QCA design methodologies is a novel concept in digital circuit design. Data security in nano-communication is crucial in data transmission and reception; hence, cryptographic approaches are necessary. The data entering the input line is encrypted by an encoder, and then sent to the designated output line, where it is decoded and transferred. The Nanorouter is offered as a data path selector, and the proposed study analyses the cell count of QCA and the circuit delay. In this manuscript, novel designs of (4:1)) Mux and (1:4) Demux designs are utilized to implement the proposed nanorouter design. The proposed (4:1) Mux design requires 3–5% fewer cell counts and 20–25% fewer area, and the propsoed (1:4) Demux designs require 75–80% fewer cell counts and 90–95% fewer area compared to their latest counterparts. The QCAPro utility is used to analyse the power consumption of several components that make up the router. QCADesigner 2.0.3 is used to validate the simulation results and output validity.

## Introduction

The advancement in VLSI technology mainly depends on scaling integrated circuits or ICs based on Moore's law. The CMOS technology is reaching its limits on the physical size through ultra-thin gate oxide effect, short channel effects, doping fluctuations and expensive lithography at the nanoscale level as significant drawbacks^1^. With considerable advances in Nanoelectronics devices, Quantum-dot cell automata (QCA) is the most encouraging option in contrast to traditional CMOS innovation. QCA device has some advantages over other by high speed, high density, and low power consumption that are very helpful for the design of digital circuits^2–4^. This technology, without any transistor, is very helpful in designing circuits at the nanoscale level. The primary unit of QCA is a cell composed of four divisions of quantum dots, with electrons located at the vertices of a square^5,6^. The QCA cell has a maximum of two electrons at a time, and these mutually stay apart at a maximum distance by repulsion through columbic force. These electrons have very little probability of being detected without an external field, so any particular electric field is required.

A router is an integral part of the internet, so it needs to be designed so that the packets coming to the input port must successfully transfer to the output port. An essential requirement is that the memory access rate should not be less than the line rate^7,8^. The QCA has the advantages of very low power dissipation, a tiny chip area and an ultra-high speed clock near the range of 1–2 THz. Using QCA technology, the router architecture is built, potentially increasing the internet speed. Cryptography is very helpful in protecting data, and this can be done very efficiently in the digital world.

For high-security communication, QCA is utilized to construct an effective Nanorouter, and by this, one can achieve less power with lesser delay and low circuit complexity. Some significant findings of this study are (1) the implementation of MUX and DEMUX through the newly proposed single-layer structure; (2) the implementation of a data path selector circuit that can work as a Nanorouter utilizing MUX and DEMUX; (3) a novel design of XOR gate having efficient area and lesser clock; (4) encoder and decoder circuit is designed using the XOR gate; (5) the power analysis of all the structure is shown and also with their thermal mapping with the help of QCA pro tool; (6) using all structure a cryptographic nano-communication architecture is obtained.

## Background

### QCA cell overview

A QCA cell is made up of four quantum dots that are implemented using metal islands on the substrate. The quantum dots adhere to the quantum confinement principle. Any QCA circuit is made up of these cells, and numerous alternative cell locations help to realise multiple logic gates. A single electron may be trapped by a dot in a cell, and it can have two electrons within it at the same time. Electrons are free and mobile, allowing them to tunnel between the dots. However, tunnelling outside the cells is not permitted due to the high potential barrier. Electrons in a cell settle along opposing diagonals due to their columbi1c interaction^9^. Two possible states are equivalent to each other; these states mainly have polarization P = − 1 and P =  + 1. The formula can define the polarization:

1 $$P = \frac{{\left( {P1 + P3} \right) - \left( {P2 + P4} \right)}}{P1 + P2 + P3 + P4}$$

**Figure 1:**
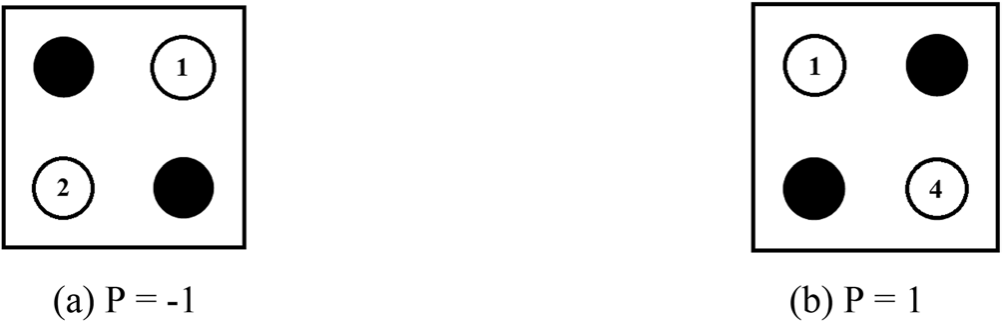
QCA cells Polarization.

The two polarization states define the binary logic states, P = − 1 will represent logic 0, and P =  + 1 will represent logic 1 (Fig. 1). A cell near the other QCA cell will polarize the other cell accordingly, and the logic will pass.

**Figure 2:**
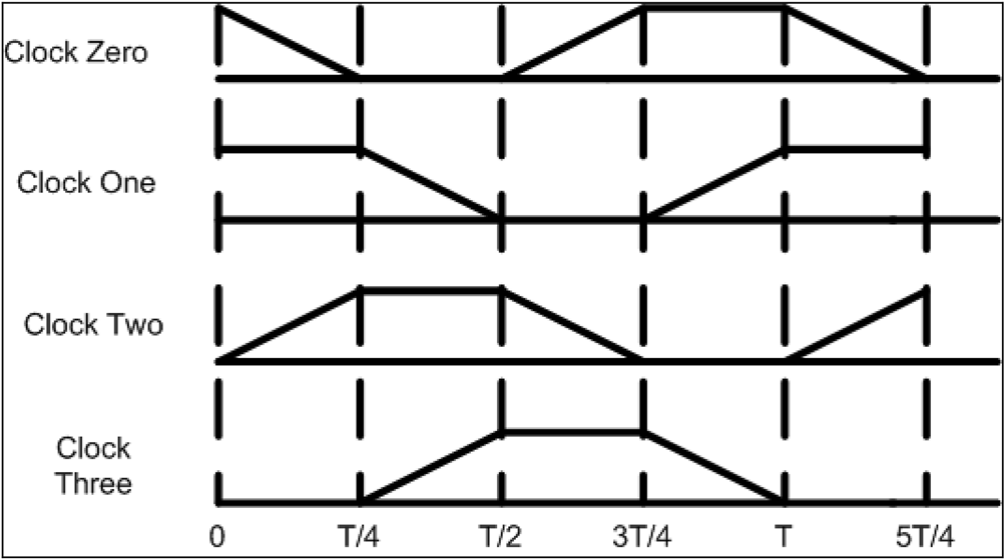
Clock zones.

The barrier between the cells can be moderated so that the logic will get passed as required, known as clocking. The clock is the only power source for a QCA cell. There are four clock phases: switch, hold, release, and relax, as shown in Fig. 2^10,11^. In the first phase, the QCA cells will get depolarized as tunnelling potential barriers are low. At this phase, the interdot barriers increase and the polarization of cells starts according to the neighbour cells. The accurate computation will happen at this phase, and the barrier remains high for further tunnelling. In phase 2, the obstacles are kept high; as a result, states will be fixed to be valid for input to the next cell. In phase 3, the barriers are lowered, and the cells are depolarized. Finally, in the last phase, the obstacles are low for the cells to remain depolarized.

There are two primary logic gates in QCA designs: inverter and majority gate. The inverter gate inverts the logic by keeping the cells diagonally to each other. As the name suggests, the majority gate gives the output by considering the majority of inputs; its production is mainly provided by *M* (*X*, *Y*, *Z*) = *XY* + *ZX* + *YZ*. The other gates can also be acquired with the help of M.

### Router architecture

**Figure 3:**
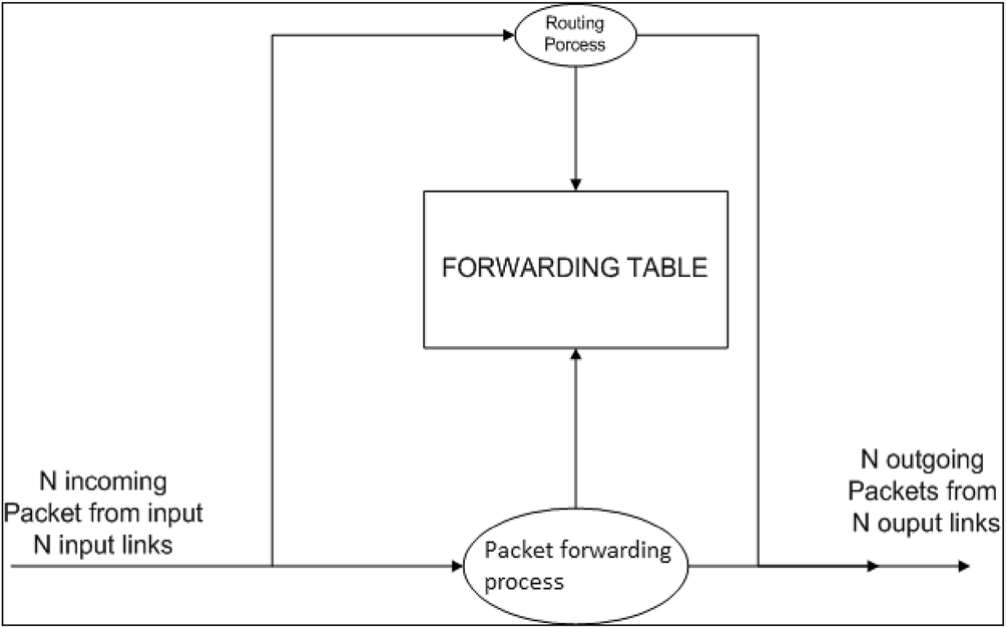
Router architecture.

It is composed of two fundamental building pieces, which are referred to as the data plane and the control plane. These blocks are based on the capabilities of a router. In this area, the control plane is responsible for mapping the network, and it is via this mapping that it is possible to execute protocols and configure the routing table. In contrast to the fact that the data plane uses a switch to forward data packets, the processing that takes place is determined by the packets themselves. The authors of this study have focused their attention on the design of data plane components^12–14^. The data plane receives information from the N incoming connections, processes it, and then sends it to the N output links. The most important part of a router is shown in Fig. 3, as seen there. At this point, the input is responsible for data linkage, search, and forwarding, and it is also the component that delivers packets to the switching fabric. The function of linking the input port and the output port is performed by the switch fabric. As shown in Fig. 3, the output port is responsible for performing the role of the reverse data link as well as receiving a packet from the switch fabric and sending that packet on to the outgoing link. It is indicated here that the routing processor is responsible for maintaining the forwarding table and carrying out activities related to network administration^15^.

When a data packet reaches the input port, it is combined with other data packets to form larger entities known as frames with the assistance of data link and physical functions. The packets also include the destination IP address, which is retrieved by the lookup table. Then, with the assistance of the forwarding table and depending on which output port is chosen by the switch fabric to forward the packets, it looks for the address that is the closest match to the maximum number of addresses in the lookup table. By executing the matching algorithm inside the routing processor, switch fabric ensures that all of the buses are linked both horizontally and vertically. There is also a period of time spent waiting for packets in the event that several packets arrive at the same time. If this occurs, one of the packets will be stopped while the other is transmitted.

## The proposed work

### XOR gate

Any digital circuit can be designed using some basic logic gates such as OR gate, AND gate, NOT gate and there are some universal gates NOR and NAND also available. In general, Exclusive-OR (XOR) and XNOR gates are utilized in designing any complex circuit.

So far, many XOR structures have been proposed in QCA technology. These are mainly based on the fundamental expression of the XOR gate as,

2 $$Y\left( {A,B} \right) = \overline{A}.B + A.\overline{B }$$

**Figure 4:**
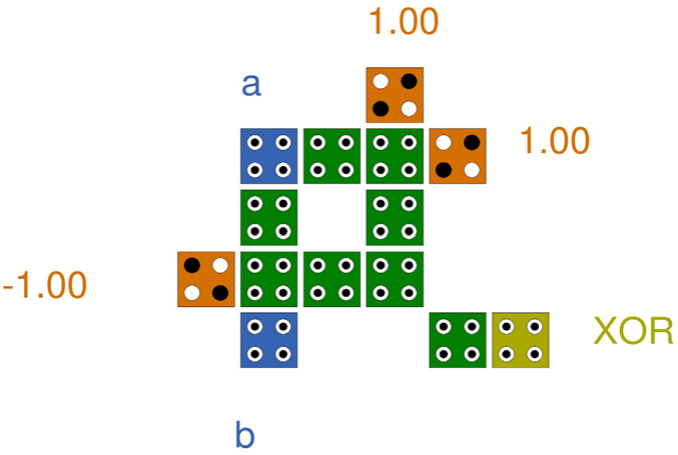
XOR gate^14^.

Equation (2) may be used to create majority-based structures. Here, in our encoder structure, an XOR gate is used to generate a cypher text by exchanging the input with the key. As a result, a more compact XOR structure is needed since the one which is utilized in the designs available is not the majority gate based and only requires 14 cells in the format^16^ (Fig. 4).

### DEMUX

A demux receives many data output lines and chooses one to be transmitted to the input lines^17^. Signalling at the selection lines enables the selection of individual output lines. With one input line and four output lines through two selection lines, the demux is located at the receiver end of the transmission line. Demux is a crucial component of the router circuit in the proposed designs. When a demux is used in a course, the direction in which data will travel may be predetermined.

**Figure 5:**
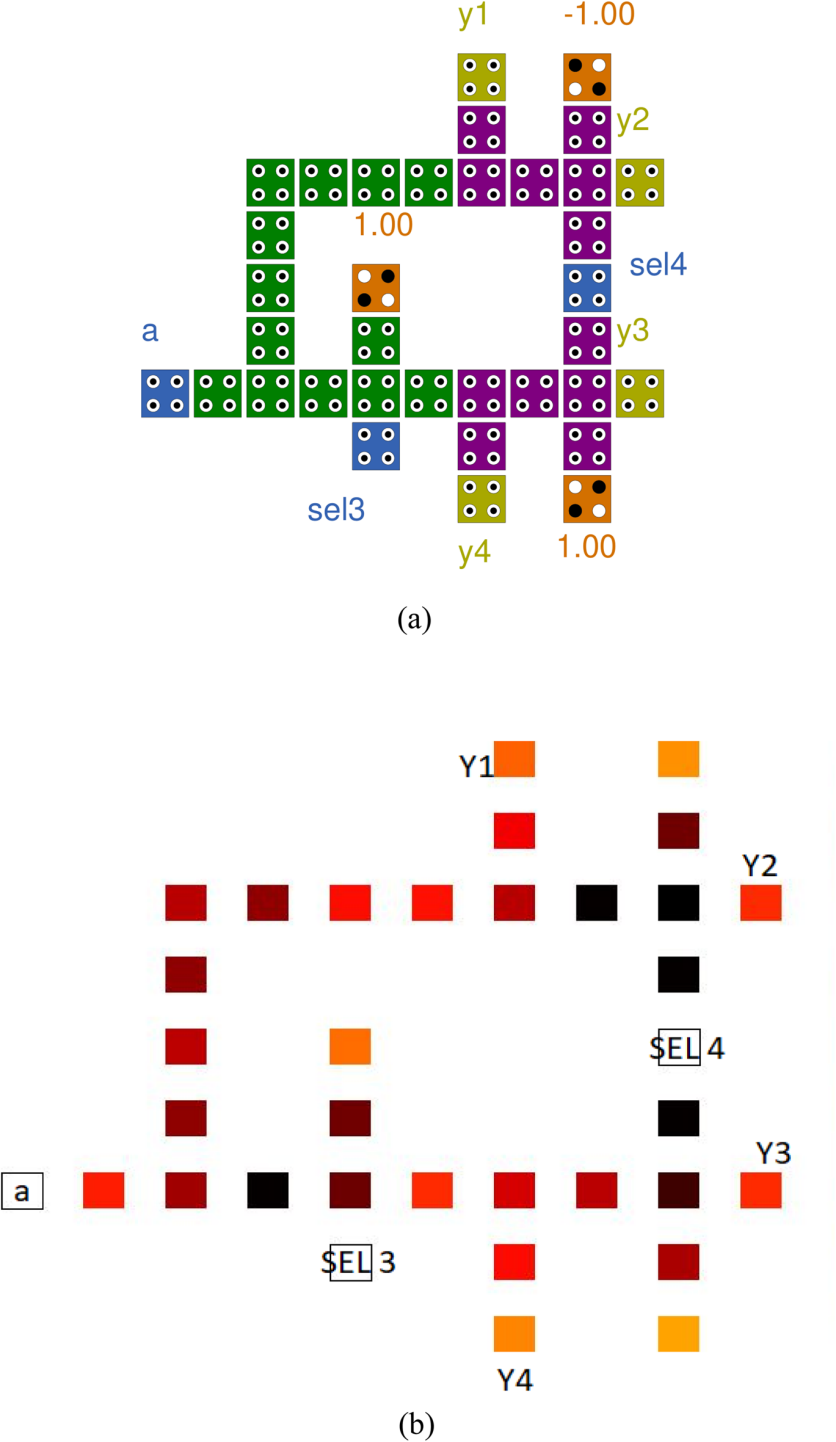
(**a**) Proposed Demux, (**b**) Thermal layout of Proposed Demux.

Work in Demux circuit designing for QCA needs to be better in the literature. Here the demux circuit designing is proposed based on the truth table, and the coplanar structure of demux is made with the majority less logic and using the concept of quantum dots. The proposed demux structure is obtained by a total number of 35 cells, as depicted in Fig. 5a and the thermal layout in Fig. 5b.

### MUX

**Figure 6:**
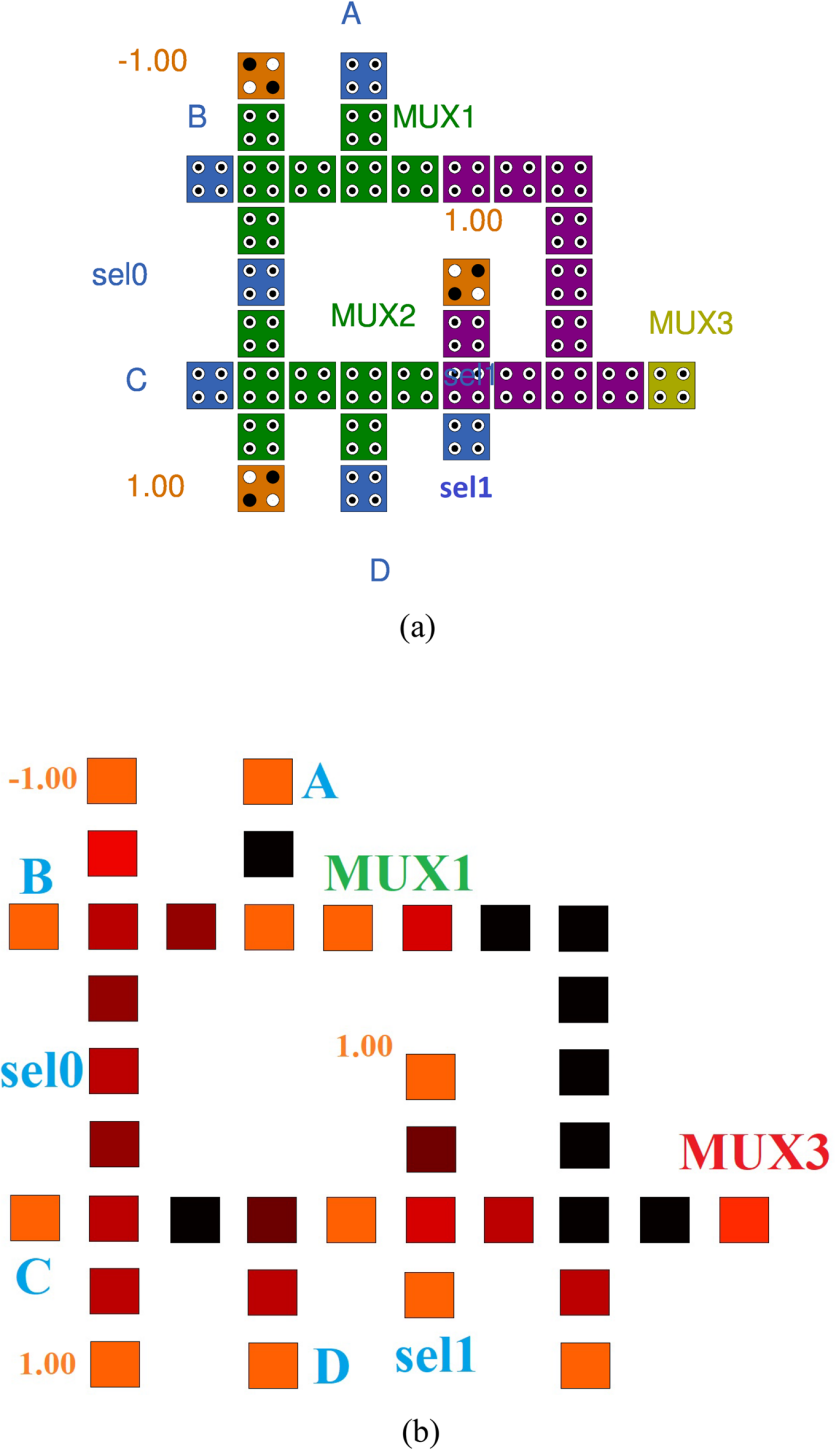
(**a**) Proposed Mux, (**b**) Thermal layout of Proposed Mux.

The MUX is used here to send part of the transmission line^18^. The 4:1 MUX is utilized in the proposed design, which has two select lines based on which the input cypher text is selected among four and one is sent. We are designing MUX with the help of the truth table available to us. Our design contains 32 cells and is also made with a majority less logic, as shown in Fig. 6a and the thermal layout in Fig. 6b.

### Proposed cryptographic architecture

**Figure 7:**
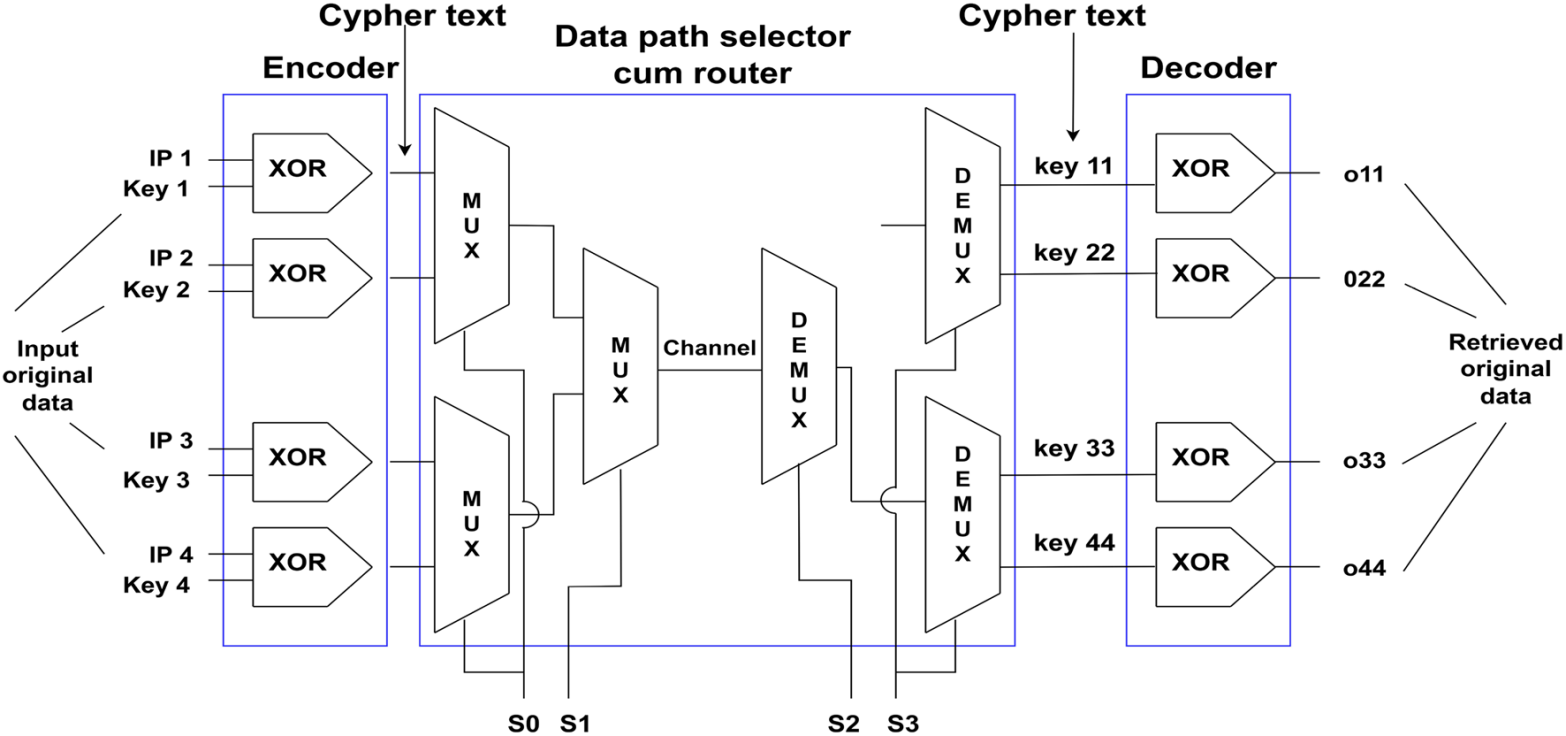
Schematic diagram of proposed nanorouter.

**Figure 8:**
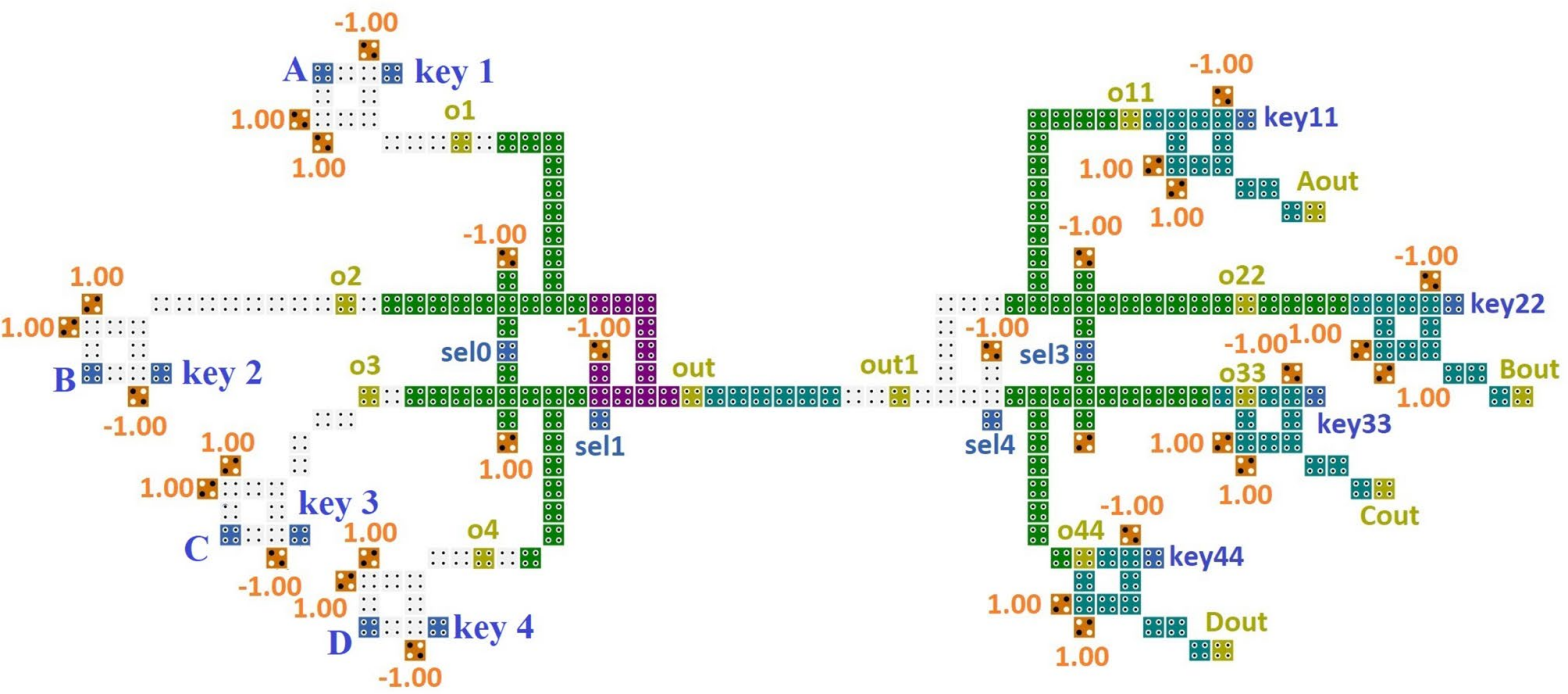
Proposed nanorouter.

The proposed design makes use of the cryptographic architecture's composition, as shown in^11,19^, and^20^. The suggested layout takes into account a large number of input ports from which the data must be sent. Next, we'll apply encryption to the incoming data to make it unreadable. The next step is for them to send encrypted data over the chosen channel to their intended recipient. Ultimately, the original message will be decrypted at the output channel. The suggested Nanorouter system's configuration and schematic diagram are shown in Figs. 7 and 8.

The router's block diagram, which is essentially a data path selector circuit, confirms this. The MUX, gearbox line and DEMUX are the three fundamental parts. In this paper, the QCA Nanorouter design is given. The channel's inputs are A, B, C, and D, while its outputs are Aout, Bout, Cout, and Dout. The MUX's select lines are 0 and 1, whereas the DEMUX's has 3 and 4. The transmission line, which consists of only the connection of QCA cells linked by distinct clock zones, carries the input channels A, B, C, and D.

Security-wise, the suggested cryptographic architecture is a good idea. The cryptographic structure in this work is a symmetric key, meaning the same key is used for both the sender and the receiver. Here, data is encrypted using an XOR gate called an encoder. Here, the keys utilized are key1, key2, and key3 on the input side, and key11, key22, key33, and key44 on the output side.

## Results

**Figure 9:**
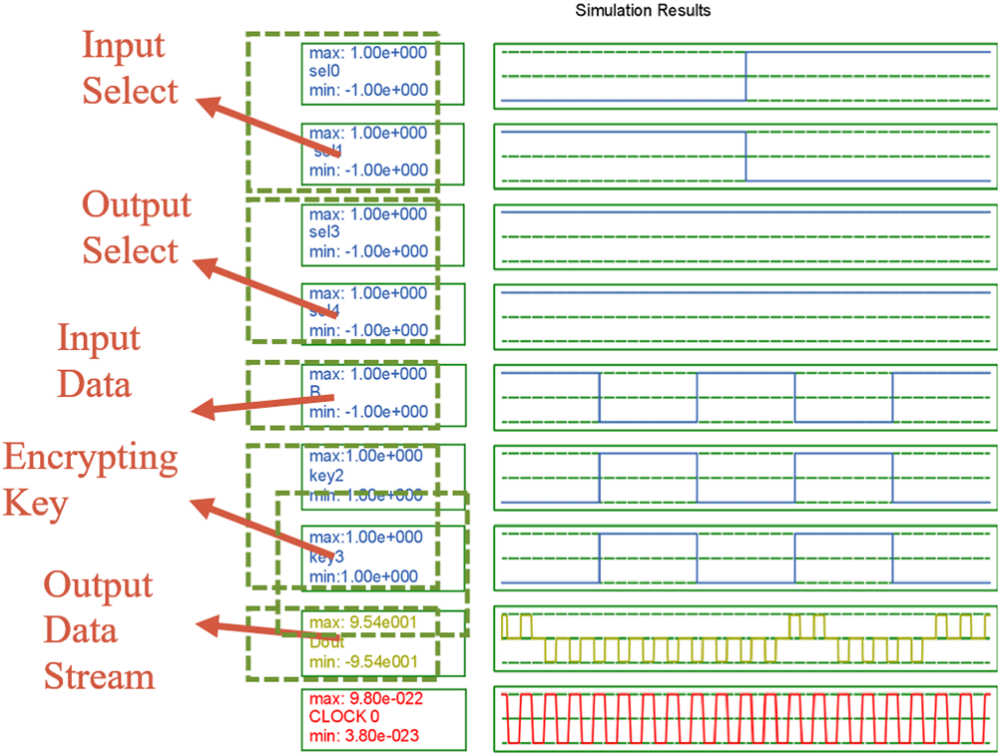
Simulation result.

The simulation result of the proposed design is shown in Fig. 9. First, the selection lines are set to Sel0, Sel1, Sel3, and Sel4 with values set as 0, 1, 1, and 1, respectively. The inputs are a = 00001111, b = 11110000, c = 00000000, d = 11111111. All the keys are the same as key = 00001111, and here the key is taken at the receiver side as same, but as there is a delay in the line and the output is received after a delay of 2 clock cycles, it is also taken after two clock cycles.

### Complexity of the circuit

Indicators of circuit complexity include the number of cells, the size of the cells, and the time it takes for a single clock cycle to complete. The complexity of the circuit is defined by the presence of an XOR gate, a 4:1 MUX, and a 1:4 DEMUX in the various modules of the proposed design, as indicated in Table 1. The suggested architecture's circuit cost is determined alongside its parts.

**Table 1:** Complexity of the proposed structure.

QCA circuits	No of cells	Total area occupied (μm^2^)	Cell area (μm^2^)	Area usage by cell (%)	Latency or clock cycle	Circuit cost (Circuit cost = total latency × total area)
XOR gate	14	0.03	0.0045	15	0.25	0.0075
Proposed MUX	32	0.029	0.0103	35.5	0.5	0.0145
Proposed DEMUX	35	0.029	0.0113	38.9	0.5	0.0145
Proposed router circuit	263	0.6	0.085	14.1	1.75	1.05

### Comparison analysis from previous works

Only a few papers on nanocommunication circuits with QCA cell implementation are available. As a consequence, the outcomes of prior work must now be compared to the projected work. Because only the old designs are accessible, the chart only mentions four earlier works. Tables 2 and 3 show that the outcome has significantly improved.

**Table 2:** Comparison between various 4:1 MUX.

References	Cell count	Latency	Area (μm^2^)
Sabbaghi-Nadooshan et al.^22^	26	0.5	0.02
Sen et al.^23^	23	0.5	0.02
Rashidi et al.^24^	15	0.5	0.01
Asfestani et al.^25^	12	0.25	0.01
AlKaldy et al.^21^	11	0.25	0.01
The proposed work (Fig. 6)	10	0.25	0.0076

**Table 3:** Comparison between various 1:4 DEMUX.

References	Cell Count	Latency	Area (μm^2^)
Shah et al.^17^	217	2	0.6
Ganesh et al.^26^	404	2	0.6
Iqbal et al.^27^	188	1	0.22
The proposed work (Fig. 5)	35	0.5	0.029

Table 2 compares the cell count, delay, and area of the proposed 4:1 MUX to earlier designs. The suggested 4:1 MUX design has 25% larger area and 1 fewer QCA cell than the most recent design presented in^21^.

Table 3 comprares the cell count, latency and area for proposed 1:4 DEMUX with previous designs. The proposed (4:1) Mux design requires 3–5% fewer cell counts and 20–25% fewer area, and the propsoed (1:4) Demux designs require 75–80% fewer cell counts and 90–95% fewer area compared to their latest counterparts as shown in Tables 2 and 3. Table 4 shows that the proposed nanorutner has 5–10% improvement in cell counts, almost 100% improvement in latency and 5–10% fewer area compared to its recent counterparts.

**Table 4:** Comparison between various nanorouters.

References	Cell count	Latency	Area (μm^2^)
Das et al.^8^	419	3.00	0.54
Sardinha et al.^28^	4026	12.00	13.81
Bikash et al.^29^	293	3.75	0.53
The proposed work (Fig. 8)	263	1.75	0.50

### Power analysis

An approximation approach like the Hartree–Fock approximation is necessary for the power consumption analysis in the QCA instance. To determine the output power, use the Hamiltonian matrix, as shown in^15^. Using the energy expenditure for a pair of neighbouring cells with opposing polarisations, as described in^15^, we can determine the average energy expenditure for a cell over the course of a clock cycle as:

3 $$E = H = \frac{\hbar }{2}.\vec{\Gamma }.\vec{\lambda }$$

Therefore, power for a single cell will be,

4 $$p_{t} = \frac{dE}{{dt}} = \frac{\hbar }{2} \cdot \frac{{d \vec{\Gamma }}}{dt} \cdot \vec{\lambda } + \frac{\hbar }{2} \cdot \vec{\Gamma }. \frac{{d\vec{\lambda }}}{dt}$$

The second term in the above equation represents the instantaneous power dissipation so that the power will be calculated by integration over time. For power dissipation analysis, the QCA Pro tool with tunnelling energy as γ = 0.5 $E_{k}$ $1\,E_{k}$ and 1.5 $E_{k}$ has been utilized, and thermal mapping of 4:1 Mux and 1:4 Demux is depicted as shown in Figs. 5b and 6b.

Here in Table 5, the power dissipation of the proposed circuits is shown, which is shown as an improved result than previous work where ${E}_{k}$ is the kink energy.

**Table 5:** Power dissipation (in meV).

Proposed designs	0.5 ${E}_{k}$	1 ${E}_{k}$	1. 5 ${E}_{k}$
4:1 Mux	92.2	114.61	153.58
1:4 Demux	69.5	81.55	97.63

**Figure 10:**
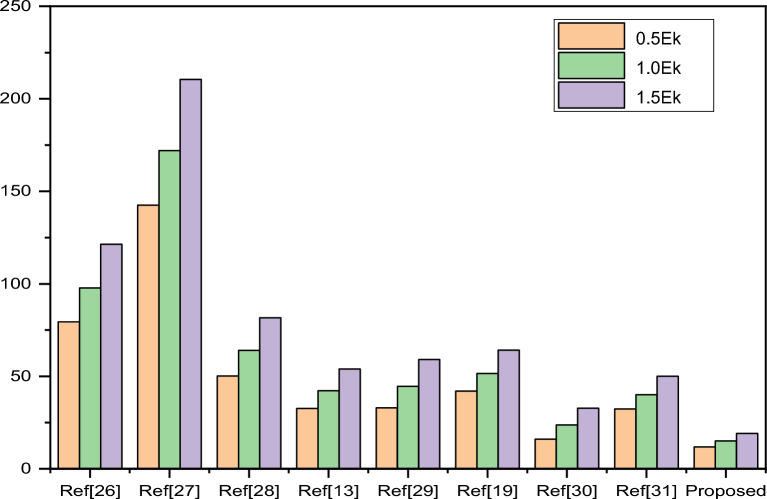
Comparison of power dissipation among various.

**Figure 11:**
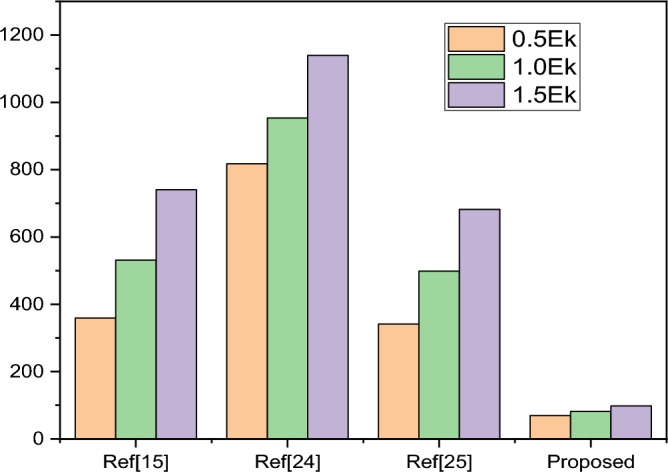
Comparison of power dissipation among various 1:4 DE MUX Structure.

Here, the power estimation of the MUX and DEMUX is analyzed, and from the table, one can easily observe that the power dissipation is significantly less in the proposed circuit designs compared to the previous methods. This happened because most gates are not utilized in the proposed plans. In Table 6, an evaluation of projected and prior circuits for various 4:1 MUX structures is performed, and the results are shown as a bar graph representation in Fig. 10, and in Table 7, a comparison of different 1:4 DEMUX structures and their results are depicted with its bar graph representation in Fig. 11.

**Table 6:** Power dissipation analysis among previous work on 4:1 MUX.

References	0.5 ${E}_{k}$	1.0 ${E}_{k}$	1.5 ${E}_{k}$
Kim et al.^28^	79.38	97.73	121.41
Teodósio et al.^29^	142.55	172.05	210.51
Amiri et al.^30^	50.16	63.96	81.65
Majeed et al.^15^	32.59	42.23	53.97
Mukhopadhyay et al.^31^	32.97	44.51	59.04
Sabbaghi-Nadooshan et al.^22^	42.01	51.53	64.12
Das et al.^32^	15.95	23.65	32.82
Das et al.^33^	32.33	40.04	50.08
Proposed work	11.82	15.06	19.09

**Table 7:** Power dissipation analysis among previous work on 1:4 DEMUX.

References	0.5 ${E}_{k}$	1.0 ${E}_{k}$	1.5 ${E}_{k}$
Shah et al.^15^	359.25	531.7	740.8
Ganesh et al.^24^	817.62	953.19	1139.54
Iqbal^25^	341.46	498.64	681.83
Proposed work	69.5	81.55	97.63

## Conclusion

Even after encryption and many other techniques implemented in communication systems, the channel is vulnerable to a variety of side-channel attacks, including timing, caching, power monitoring, and electromagnetic assaults. Because the QCA-based circuit uses minimal power, it is not vulnerable to power analysis attacks. In this way, the proposed circuits are also resistant to comparable attacks. The recommended encoder, which uses an XOR, encrypts the input 8-bit data with the 8-bit key before delivering it to the output, where it is decrypted by the decoder, which also uses the 8-bit key. This, however, applies to any message size. When compared to the newest existing designs, the suggested Nanorouter circuit needs 8–10% fewer cell counts, 50–60% less latency, and over 3–5% less total space, thus demonstrating that the proposed design exceeds the current best-in-class designs. The Nanorouter circuit proposed here will be widely employed in future cryptographic algorithm implementations in QCA-based secure systems.

## Data Availability

The datasets generated during and/or analysed during the current study are available from the corresponding author on reasonable request.
